# Adenosquamous carcinoma of the ampulla of Vater - a rare disease at unusual location

**DOI:** 10.1186/1477-7819-11-124

**Published:** 2013-05-31

**Authors:** Shang-Ju Yang, Chun-Hsiang Ooyang, Shang-Yu Wang, Yu-Yin Liu, I-Min Kuo, Chien-Hung Liao, Ting-Jung Wu

**Affiliations:** 1 Department of Traumatology and Emergency Surgery, Chang-Gung memorial hospital, Linkou Fu-Shin Street, Taoyuan 333, Taiwan; 2 Department of General Surgery, Chang-Gung memorial hospital, Linkou Fu-Shin Street, Taoyuan 333, Taiwan

## Abstract

Adenosquamous carcinoma is defined as a tumor in which both glandular and squamous elements are histologically malignant. Although some published studies have analyzed and discussed adenosquamous carcinomas, hybrid malignancy of the ampulla of Vater has rarely been discussed thus far in the literature. In this study, we report the case of a 64-year-old man who presented with jaundice and intermittent abdominal dull pain that persisted for several weeks. The patient was diagnosed with adenosquamous carcinoma of the ampulla of Vater and underwent pancreaticoduodenectomy. The final diagnosis was adenosquamous carcinoma of the ampulla of Vater, T3N1M0, stage IIB. Although R0 resection was performed, he had multiple liver metastases 2 months after the operation; he died 4 months later. Upon reviewing the medical records of our institute, we identified 4 patients who were diagnosed with adenosquamous carcinoma of the ampulla of Vater in the past 2 decades. We also identified only five reported cases of this lesion in the English literature. Adenosquamous carcinoma of the ampulla of Vater is a rare disease with a dismal prognosis. Surgical intervention does not appear to prolong patient survival. Early recurrence and distal metastasis may be encountered after surgery.

## Background

Adenosquamous carcinoma (ASC) is defined as a tumor in which both glandular and squamous elements are histologically malignant. The mixed tumor was first reported in 1907. Its occurrence is more common in areas where adenocarcinomas arise frequently, such as the stomach, intestine, and uterus. ASC has also been identified in the esophagus, anus, and vagina, where squamous cell carcinomas predominate. Although some published studies have analyzed and discussed this condition, ASC of the ampulla of Vater (AmV) has rarely been discussed in the literature. In this report, we present a case of ASC of the AmV and review the experience of this rare disease in our institute.

## Case presentation

A 64-year-old man presented with jaundice and intermittent abdominal dull pain that persisted for several weeks. The only findings on physical examination were icteric sclera and yellowish skin. No tenderness or palpable mass was detected in his abdomen. On admission, the laboratory examination revealed a serum total bilirubin level of 6.4 mg/dL, aspartate aminotransferase level of 19 IU/L, alanine aminotransferase level of 21 IU/L, and alkaline phosphate level of 229 IU/L. All tumor marker levels were normal. Contrast-enhanced computed tomography (CT) revealed a protruding tumor in the AmV without invasion into adjacent organs. There were several enlarged regional lymph nodes. There was no distal metastatic lesion in the lungs, bone, or liver. The major vessels were free of this tumor. This patient underwent endoscopic retrograde cholangiopancreatography and a concurrent biopsy, and the histopathological diagnosis was of a poorly differentiated adenocarcinoma of the AmV, tumor, node, metastis (TNM) classification T2, N1, M0, stage IIA. The patient underwent standard pancreaticoduodenectomy (PD) with lymph node dissection.

**Figure 1:**
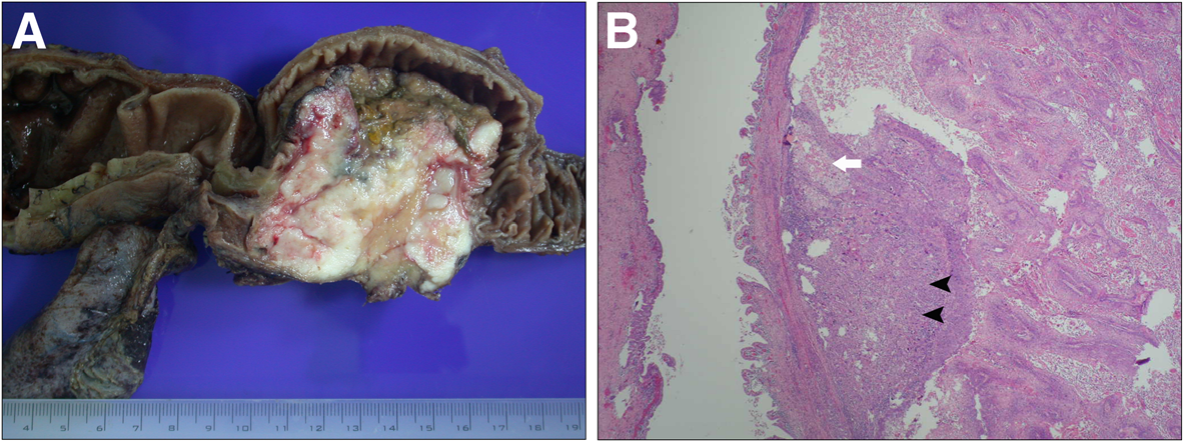
**The gross and microscopic pictures of adenosquamous carcinoma of ampulla of Vater.** (**A)** The specimen was a polypoid tumor in the ampulla of Vater that measured 3.4 cm × 2.2 cm with pancreatic invasion. (**B**) Microscopically, the tumor comprised squamous cell carcinoma (arrowhead) with some nets of adenocarcinoma (arrow).

**Figure 2:**
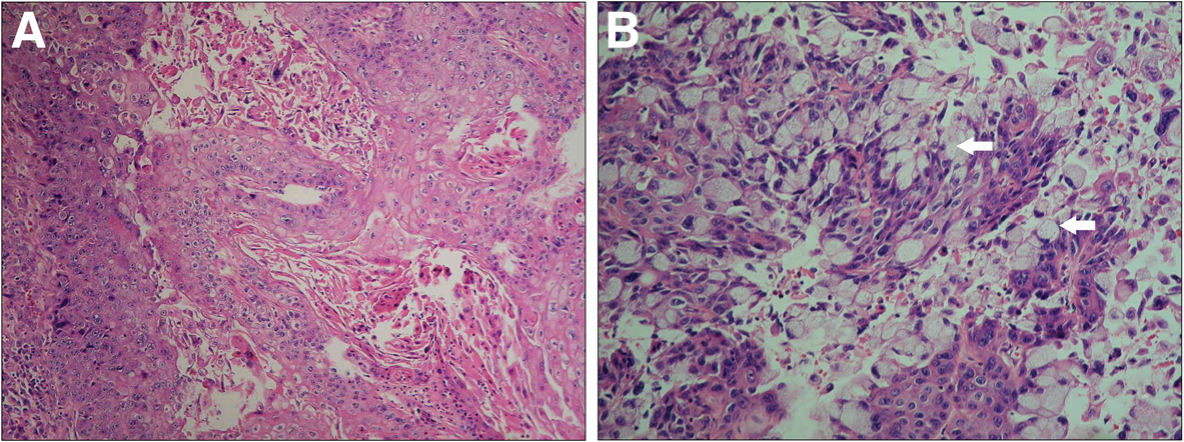
**The mixed histologic presentations of adenosquamous carcinoma of ampulla of Vater.** (**A**) In a high-power field, the majority of the tumor was composed of moderately differentiated squamous cell carcinoma. (**B**) Several focal signet ring-like glandular adenocarcinoma components (arrow) were present in this tumor.

On gross examination, a polypoid tumor, measuring 3.4 cm × 2.2 cm, with invasion to the pancreas was detected in the AmV (Figure 1A). Microscopically, the tumor comprised both malignant squamous and glandular components (Figure 1B). The majority of the tumor was composed of a moderately differentiated squamous cell carcinoma (Figure 2A). Several focal signet ring-like glandular adenocarcinoma components were confined in the AmV (Figure 2B). The resection margins were uninvolved, and regional lymph node involvement was identified in 2/46 retrieved nodes. The final diagnosis was ASC of the AmV, T3, N1, M0, stage IIB. The patient’s postoperative recovery was uneventful, and he was discharged 2 weeks after surgery. He was followed up 1 week after discharge and then regularly every 2 weeks thereafter. Routine biochemical and tumor markers were examined monthly, but no obvious change was observed. Adjuvant chemotherapy as Gemcitabine 1000 mg/m^2^ per month combined with local radiation was arranged for this patient 6 weeks after the operation. Follow-up CT was scheduled for 3 months after surgery, but the patient presented with progressive jaundice 2 months after surgery. Abdominal CT revealed multiple metastatic nodules in the liver. This patient received palliative chemotherapy as Gemcitabine and Cisplatin once after the tumor progression. Nevertheless, his condition deteriorated, and the tumor progressed rapidly. He died 6 months after surgery.

## Discussion

The histogenesis of ASC remains uncertain. Four hypotheses regarding its histogenesis are summarized as follows: (1) pluripotent epithelial stem cells capable of inducing the malignant transformation of both cell types; (2) squamous metaplasia in the intestinal mucosa; (3) adenocarcinoma transforming into squamous cell carcinoma; and (4) collision of both malignant tumors. Although we do not understand the histogenesis of ASC, it exhibits a more aggressive biologic behavior and is associated with a worse prognosis than conventional adenocarcinoma according to previous reports.

Four cases of ASC of the AmV have been reported in the malignancy registry in our institute in the past two decades. Compared to adenocarcinoma, ASC of the AmV is an uncommon malignancy. The most common presentation of the patients in this series was jaundice with intermittent right upper quadrant pain. All of the patients received surgical interventions. Three patients underwent PD, and one underwent ampullectomy. The demographic characteristics and the American Joint Committee on Cancer (AJCC) 7^th^ tumor stage classification of these patients are listed in Table 1. Preoperative diagnosis was difficult because of the lack of defining characteristics in imaging studies and the difficulty in acquiring both malignant components by limited biopsy. All of the patients had a dismal prognosis, and most of them had early distal metastasis after surgery. The median survival was 8.5 months (range 6 to 14 months).

**Table 1:** Characteristics of patients with adenosquamous carcinoma of the ampulla of Vater

**Case number**	**Gender**	**Age (years)**	**Initial symptoms**	**AJCC 7**^**th **^**TNM stage**
1	Male	82	Jaundice	T2, N0, M0, stage IB
2	Male	68	RUQ pain and jaundice	T4, N1, M0, stage III
3	Female	34	RUQ pain and jaundice	T4, N0, M0, stage III
4	Male	77	RUQ pain and jaundice	T3, N1, M0, stage IIB

AJCC, American Joint Committee on Cancer; RUQ, right upper quadrant of the abdomen; TNM, tumor, node, metastasis.

A review of the English literature revealed only five reported cases of ASC of the AmV. We have summarized these reports, including the clinical course and final prognosis of ASC of the AmV, in Table 2[1-4]. ASC of the AmV is a virulent disease with a worse prognosis than that of adenocarcinoma. A majority of patients with ASC of the AmV are managed with operative resection. Surgery remains the mainstay therapy for this disease. A review of the data revealed that the most common procedure performed was PD followed by ampullectomy. Surgical interventions do not appear to improve patient survival. Most patients with ASC of the AmV experienced early distal metastasis and short survival after surgery. Our limited experience makes it difficult to determine the clinical course of this disease and the efficacy of surgical interventions. Because of the specificity of anatomic structures, PD or ampullectomy is necessary for complete resection of ASC of the AmV. Although these procedures have been performed with a remarkably low mortality rate (<4%), the incidence of postoperative morbidity can be as high as 30 to 40%. It is hard to draw a conclusion on whether performing major surgery in these patients is beneficial, because of the potentially high morbidity. One report presented two patients with prolonged survival (19 and 46 months after surgery) [3]. As noted by Lee *et al*. [3], complete surgical resection may prolong patient survival; however, this finding was not supported by other reports [1,2]. Because of findings of early recurrence and metastasis after surgery, micrometastasis may have occurred, but we could not detect it prior to surgery. Currently, there is no established diagnostic tool for detecting micrometastasis. Positron emission tomography may be another diagnostic tool for detecting micrometastasis, but the efficacy of this tool requires more evidence for confirmation.

**Table 2:** Reports of adenosquamous carcinoma of the ampulla of Vater

**Study**	**Case**	**Age (years)/gender**	**Stage**	**Management**	**Postoperative distal metastasis**	**Survival**	**Overall survival (months)**
Ueno *et al*. [1]	1	47/M	IIB	PD	+	Dead	10
Ri *et al*. [2]	2	62/F	IIA	PPPD	+	Dead	11
Lee *et al*. [3]	3	48/M	IB	PPPD	-	Alive	19
	4	80/F	IIB	PPPD	-	Alive	46
Song *et al*. [4]	5	76/M	NM	Stenting	+	Dead	8
Present study	6	82/M	IB	Ampullectomy	+	Dead	14
	7	68/M	III	PD	+	Dead	7
	8	34/F	III	PD	+	Dead	10
	9	77/M	IIB	PD	+	Dead	6

M, male; F, female; NM, not mentioned; PD, pancreaticoduodenectomy; PPPD, pylorus-preserving pancreaticoduodenectomy.

Some authors have suggested conservative management of patients with ASC of the AmV diagnosed before surgery, to reduce postoperative morbidity and achieve comparable survival as observed in patients undergoing surgery [4]. Although there was no published report evaluating the efficacy of chemoradiation against this disease, in view of experience of ASC at other locations, we believed that chemoradiation is the first choice of treatment for ASC of the AmV if we can make the diagnosis before the operation. However, the correct pre-operative histologic diagnosis was difficult to determine. We reviewed all of the biopsy specimens with ASC of the AmV, but none of them exhibited patterns typical of both malignant components. Follow-up should be more frequent to detect possible early recurrence and distal metastasis in patients with ASC of the AmV.

## Conclusions

In conclusion, ASC of the AmV is a rare disease with a dismal prognosis. Its symptoms are similar to those of adenocarcinoma of the AmV, and accurate preoperative diagnosis is difficult. Surgical interventions do not appear to prolong patient survival, and early recurrence and distal metastasis may be encountered after surgery. However, the rarity of this malignancy makes it difficult to draw firm conclusions regarding its clinical course and overall prognosis.

## Consent

Written informed consent was obtained from the patients or the next of kin of the patients for publication of this report and any accompanying images. Copies of the written consent forms are available for review by the Editor-in-Chief of this journal.

### Permissions

None of the material has been previously published.

## Abbreviations

AJCC: American Joint Committee on Cancer; AmV: Ampulla of Vater; ASC: Adenosquamous carcinoma; CT: Computed tomography; PD: Pancreaticoduodenectomy; PPPD: Pylorus-preserving pancreaticoduodenectomy.

## Competing interests

The authors declare that they have no competing interests.

## Authors’ contributions

S-J Y, S-Y W, and Y-Y L performed the surgical treatment and drafted the manuscript. C-S O and I-M K revised the registry record and performed the pathological studies. T-J W revised this manuscript. C-H L critically reviewed the manuscript and gave final approval for publication. All authors have read and approved the final manuscript.
